# Systems biology combining human- and animal-data miRNA and mRNA data identifies new targets in ureteropelvic junction obstruction

**DOI:** 10.1186/s12918-017-0411-7

**Published:** 2017-03-01

**Authors:** Theofilos Papadopoulos, Audrey Casemayou, Eric Neau, Benjamin Breuil, Cécile Caubet, Denis Calise, Barbara A. Thornhill, Magdalena Bachvarova, Julie Belliere, Robert L. Chevalier, Panagiotis Moulos, Dimcho Bachvarov, Benedicte Buffin-Meyer, Stéphane Decramer, Françoise Conte Auriol, Jean-Loup Bascands, Joost P. Schanstra, Julie Klein

**Affiliations:** 1 Institut National de la Santé et de la Recherche Médicale (INSERM), U1048, Institute of Metabolic and Cardiovascular Diseases–I2MC, 1 avenue Jean Poulhès, B.P. 84225, 31432 Toulouse Cedex 4, France; 2 Université Toulouse III Paul-Sabatier, Toulouse, France; 3 Department of Pediatrics, University of Virginia School of Medicine, Charlottesville, VA USA; 4 Department of Molecular Medicine, Université Laval, Québec, Canada; 5 Centre de recherche du CHU de Québec, L’Hôtel-Dieu de Québec, Québec, Canada; 6 HybridStat Predictive Analytics, Aiolou 19, 10551 Athens, Greece; 7 Institute of Molecular Biology and Genetics, Biomedical Sciences Research Center ‘Alexander Fleming’, Fleming 34, 16672 Vari, Greece; 8 Service de Néphrologie–Médecine Interne–Hypertension Pédiatrique, CHU Toulouse, Hôpital des Enfants, 31059 Toulouse, France; 9 Centre De Référence des Maladies Rénales Rares du Sud Ouest (SORARE), 31059 Toulouse, France; 10 Unité de recherche clinique pédiatrique, Module plurithémathique pédiatrique du Centre d’Investigation Clinique Toulouse 1436 Hôpital des enfants 330 avenue de grande bretagne, 31059 Toulouse, France; 11 DéTROI–Inserm U1188–Université de La Réunion, Diabète athérothrombose Thérapies Réunion Océan Indien, CYROI, 2, rue Maxime Rivière, 97490 Sainte Clotilde, La Réunion France

**Keywords:** Obstructive nephropathy, miRNAs/microRNAs, Microarrays, let-7a-5p and miR-29b-3p, DTX4 and NAV1

## Abstract

**Background:**

Although renal fibrosis and inflammation have shown to be involved in the pathophysiology of obstructive nephropathies, molecular mechanisms underlying evolution of these processes remain undetermined. In an attempt towards improved understanding of obstructive nephropathy and improved translatability of the results to clinical practice we have developed a systems biology approach combining omics data of both human and mouse obstructive nephropathy.

**Results:**

We have studied in parallel the urinary miRNome of infants with ureteropelvic junction obstruction and the kidney tissue miRNome and transcriptome of the corresponding neonatal partial unilateral ureteral obstruction (UUO) mouse model. Several hundreds of miRNAs and mRNAs displayed changed abundance during disease. Combination of miRNAs in both species and associated mRNAs let to the prioritization of five miRNAs and 35 mRNAs associated to disease. In vitro and in vivo validation identified consistent dysregulation of let-7a-5p and miR-29-3p and new potential targets, E3 ubiquitin-protein ligase (DTX4) and neuron navigator 1 (NAV1), potentially involved in fibrotic processes, in obstructive nephropathy in both human and mice that would not be identified otherwise.

**Conclusions:**

Our study is the first to correlate a mouse model of neonatal partial UUO with human UPJ obstruction in a comprehensive systems biology analysis. Our data revealed let-7a and miR-29b as molecules potentially involved in the development of fibrosis in UPJ obstruction via the control of DTX4 in both man and mice that would not be identified otherwise.

**Electronic supplementary material:**

The online version of this article (doi:10.1186/s12918-017-0411-7) contains supplementary material, which is available to authorized users.

## Background

Congenital obstructive nephropathy is the main cause of end stage renal disease (ESRD) in children [1]. This contrasts sharply with adult ESRD, which for the greater part originates from type II diabetes and hypertension [2]. The most frequent cause of congenital urinary tract obstruction is ureteropelvic junction (UPJ) obstruction with an estimated incidence of one in 1000–1500 [3]. The spectrum of renal abnormalities varies greatly in UPJ obstruction ranging from subtle changes such as modified proximal or tubular size, chronic tubulointerstitial injury, glomerulosclerosis, fibrosis, aberration of nephron development and in severe cases (less than 1%) renal dysplasia [4]. The gold standard in diagnosis of UPJ is by prenatal ultrasonography with subsequent evaluation in the postnatal period [5]. However, this method is not sensitive enough to accurately estimate renal function and functioning nephron number [6]. This has led to an urgent need for the development of biomarkers to assess the severity of UPJ obstruction and to help the clinicians to decide if and when pyeloplasty is required [5, 7].

Due to the fact that limited human kidney samples are available, almost all observations on the pathophysiology of UPJ obstruction are based on animal models, which potentially limit the transferability of the observations in the clinical context. As a consequence, the pathophysiological mechanisms of UPJ obstruction remain incompletely understood. Renal lesions in UPJ obstruction have been described including tubular proliferation/apoptosis, renin-angiotensin system activation, inflammation, and fibrosis [4, 5, 8, 9]. Interstitial fibrosis is a late consequence of congenital UPJ obstruction, and can be attenuated by early release of obstruction, but not if nephron number is reduced [10]. It is becoming increasingly clear that patients with congenital urinary tract obstruction must be followed into adulthood, as the lesions can progress over the entire life [2].

MicroRNAs (miRNAs) are small non-coding RNAs (20–24 nt) that regulate gene expression by blocking the translation of proteins and are involved in multiple molecular pathways and pathologies. MiRNAs are now considered promising molecules for biomarkers and/or targeted therapy of disease [11–14]. While, kidney diseases are no exception [15, 16], to our knowledge no studies specifically report miRNAs related to UPJ obstruction. There is evidence connecting dysregulated miRNAs including miR-21 and miR-29 with kidney fibrosis [17–22], an important feature in severe UPJ obstruction. Moreover, knock-down of DICER (the main protein involved in the biogenesis of miRNAs [23, 24]) leads to congenital anomalies of the kidney and the urinary tract (CAKUT) in mice [25]. MiRNAs are very stable in urine, a biofluid which can be collected non-invasively and can be valuable source of molecules to monitor diseases of the kidney and urinary tract [16]. These observations suggest that studying miRNAs in UPJ obstruction might help to understand the development of obstructive nephropathy and/or provide early markers of pathological obstruction.

In this study, we analysed the modification of urinary miRNAs in UPJ obstruction. To improve upon the clinical translatability of our results and compensate for the lack of tissue availability in human disease, we combined miRNA data obtained in human urine and miRNA and mRNA data in kidney tissue of a neonatal partial unilateral ureteral obstruction (UUO) mouse model. This combined systems biology-based approach followed by an in vitro validation pointed to the consistent dysregulation of specific miRNAs, let-7a-5p and miR-29-3p and to new potential targets, E3 ubiquitin-protein ligase (DTX4) and neuron navigator 1 (NAV1) in UPJ obstruction that would not be identified otherwise.

## Results

### MiRNA abundance changes in urine of newborns with UPJ obstruction

A total of 20 male UPJ obstruction patients and eight male healthy age-matched (<1 year-old) individuals were studied (Table 1). The severity of the obstruction varied with hydronephrosis grades from 1 to 4 and pelvic dilatation sizes from 6 to 40 mm (Table 1). We compared the urinary miRNA abundance of UPJ obstruction patients to urine of healthy controls using microarray analysis. This yielded the identification of 227 miRNAs with changed urinary abundance between the two groups (unadjusted *p* < 0.05, Additional file 1).

**Table 1:** Clinical data of the human UPJ obstruction patients

	*N*	Age (days) at urine sampling *median* [range]	HN grade *median* [range]	Pelvic diameter mm *median* [range]
Healthy controls	8	112 [20–201]	n.a.	n.a.
UPJ obstruction	20	74 [3–269]	2 [1–4]	15 [6–40]

*HN* hydronephrosis, *n.a.* not applicable

### MiRNA and mRNA expression changes in renal tissue of a neonatal partial UUO mouse model

The renal miRNA and mRNA profiles of nine neonatal mice with partial UUO (hydronephrosis grades 2 and 3) and nine control sham operated mice were studied using microarray analysis (Table 2). This led to the identification of 79 differentially expressed miRNAs and 706 differentially expressed mRNAs, respectively (unadjusted *p* < 0.05, Additional files 2 and 3).

**Table 2:** Experimental data of the partial UUO model animals

	*N* (Male/Female)	HN grade *median* [range]	Pelvic diameter in mm *median* [range]
Sham	9 (4/5)	n.a.	1.2 [0.8–1.5]
Partial UUO	9 (6/3)	2.5 [2–3]	1.5 [1–2.8]

*HN* hydronephrosis, *n.a.* not applicable

### Identification of most prominent dysregulated miRNAs commonly associated with obstructive nephropathy in humans and mice

To prioritize the molecular features with potentially the highest impact on the development of kidney lesions, we next identified the miRNAs that could consistently reflect the human disease by comparing the similarity of the human (urine) and animal (kidney tissue) miRNA signature taking advantage from the fact that miRNAs are well conserved between species [26]. The 227 differentially expressed human miRNAs and 79 differentially expressed mouse miRNAs were combined. This led to the identification of 18 common miRNAs (Additional file 4) that were reduced to five miRNAs when applying a fold change threshold of 2.5 in mice tissue. These five miRNAs were let-7a-5p miR-16-5p, miR-29b-3p, miR-125b-5p and miR-26a-5p (Table 3). Correlation of the urinary abundance of the five miRNAs in UPJ patients with clinical parameters showed that miR-125-5p was inversely correlated with hydronephrosis grade (spearman *r* = −0,63, *p* = 0,003, Table 4). In addition, a slight but significant inverse correlation with pelvic diameter was also observed for miR-let-7a-5p and miR-125-5p and with hydronephrosis grade for miR-let-7a-5p and miR-26a-5p, and a slight but significant positive correlation with age for miR-let-7a-5p and miR-16-5p (Table 4).

**Table 3:** Most prominent dysregulated miRNAs commonly associated to the partial UUO model and human UPJ obstruction

miRNA	UPJ vs Healthy (urine)		Partial UUO vs Sham (kidney)	
	FC	*p*-value	FC	*p*-value
let-7a-5p	−1,559	0,003	−3,558	0,004
miR-16-5p	−1,293	0,002	−2,913	0,0007
miR-29b-3p	−1,153	0,03	10,073	3,10E-08
miR-125b-5p	−1,18	0,03	−3,219	0,003
miR-26a-5p	−1,376	0,03	−3,175	0,001

*FC* fold change

**Table 4:** Correlation of the urinary abundance of the five miRNAs in UPJ patients with clinical parameters

	Pelvic diameter (mm)		Hydronephrosis grade		Age (days)	
	Spearman r	*p*-value	Spearman r	*p*-value	Spearman r	*p*-value
hsa-let-7a-5p	−0,47	0,04	−0,54	0,01	0,53	0,02
hsa-miR-125b-5p	−0,47	0,04	−0,63	0,003	0,26	n.s.
hsa-miR-26a-5p	−0,33	n.s.	−0,50	0,02	0,07	n.s.
hsa-miR-16-5p	−0,20	n.s.	−0,18	n.s.	0,49	0,03
hsa-miR-29b-3p	0,40	n.s.	0,37	n.s.	−0,29	n.s.

### Identification of most prominent dysregulated pathways and mRNA targets in obstructive nephropathy

Seven hundred six differentially expressed mRNAs were observed in kidneys of neonatal mice with partial UUO, including increased expression of markers of fibrosis such as Tgf-β1 and different forms of collagen (Additional file 3). To prioritize the mRNA targets, the five selected common miRNAs in mice and human obstructive nephropathy were combined with these 706 differentially expressed mRNAs using Ingenuity Pathway Analysis (IPA).

Then, we focused on the direct connections of the five miRNAs as generated from the predicted networks from IPA analysis. This led to the identification of 35 predicted target mRNAs for these five miRNAs (Additional files 5, 6, 7 and 8). Next, in order to focus on the most prominent molecular changes, only mRNAs predicted to be targeted by at least two of the five miRNAs were kept for further analysis: E3 ubiquitin-protein ligase DXT4 (Dtx4), leiomodin-1 (Lmod1), a disintegrin-like and metallopeptidase (reprolysin type) with thrombospondin type 1 motif, 19 (Adamts19) and neuron navigator 1 (Nav1) (Table 5, Additional file 5).

**Table 5:** Most prominent deregulated mRNA targets in obstructive nephropathy

Gene symbol Description	FC	*p*-value	let-7a-5p	miR-125b-5p	miR-16-5p	miR-26a-5p	miR- 29b-3p
*Dtx4*							
E3 ubiquitin-protein ligase or deltex 4 homolog (Drosophila)	2,41	0,001	√	√			
*Lmod1*							
Leiomodin 1 (smooth muscle)	1,57	0,001		√	√		
*Adamts19*							
A disintegrin-like and metallopeptidase (reprolysin type) with thrombospondin type 1 motif, 19	2,97	0,0006				√	√
*Nav1*							
Neuron navigator 1	1,57	0,002				√	√

*FC* fold change

### MiRNA knockdown leads to dysregulated expression of the mRNA targets in renal cells in vitro

**Fig. 1:**
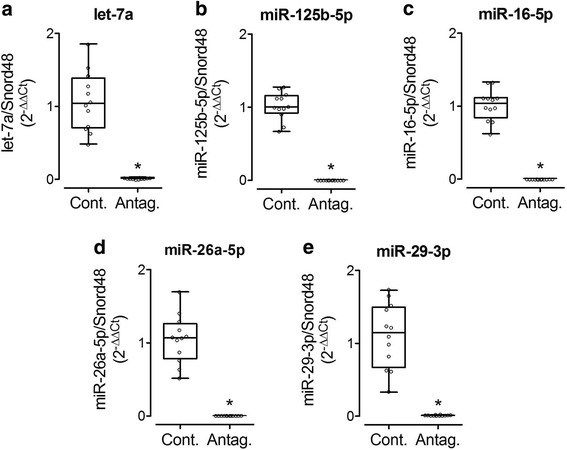
Selected miRNA expression in response to antagomirs in vitro. Expression of let-7a (**a**), miR-125b-5p (**b**), miR-16-5p (**c**), miR-26a-5p (**d**) and miR-29b-3p (**e**) was assessed by RT-PCR in HK2 cells treated or not with antagomirs. Cont: control; Antag: antagomir. *p < 0.05 versus Cont

To validate the results of the in silico analysis, we next assessed whether the predicted miRNA-mRNA target pairs were directly associated in human renal cells. Human kidney cells (HK2) were treated for 48 h with chemically modified molecules blocking the action of specific miRNA (antagomirs). In the presence of antagomirs, the detected signal of let-7a, miR-16-5p, miR-125b-5p, miR-26a-5p and miR-29b-3p was significantly decreased (Fig. 1).

**Fig. 2:**
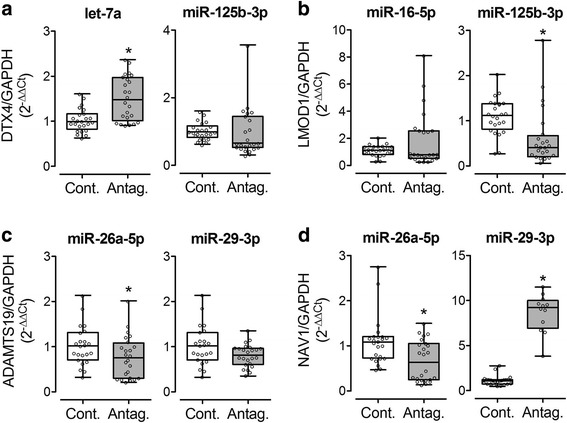
Selected mRNA predicted target expression in response to antagomirs in vitro. Expression of E3 ubiquitin-protein ligase DXT4 (DTX4) (**a**), leiomodin-1 (LMOD1) (**b**), a disintegrin-like and metallopeptidase (reprolysin type) with thrombospondin type 1 motif, 19 (ADAMTS19) (**c**) and neuron navigator 1 (NAV1) (**d**) was assessed by RT-PCR in HK2 cells treated or not with antagomirs against let-7a, miR-125b-5p, miR-16-5p, miR-26a-5p or miR-29b-3p. Cont: control; Antag: antagomir. *p < 0.05 versus Cont

MRNA expression of let-7a and miR-125b-3p target *DTX4* was significantly increased in response to downregulation of let-7a but was unmodified by antagomir anti-miR-125b-3p (Fig. 2a). Moreover, significant upregulation of neuron navigator 1 *NAV1* was observed in HK2 cells treated with the miR-29b-3p antagomir (Fig. 2d). Surprisingly, the use of antagomirs for miR-125b-5p and miR-26a-5p resulted in slight but significant downregulation of *LMOD1*, *ADAMTS19* and *NAV1* (Fig. 2b–d), a result opposite than the predicted regulation, which possibly indicates an indirect mechanism of effect of these two miRNAs on these targets. Antagomirs for miR-125b-5p, miR-16-5p and miR-29b-3p showed no effect on *DTX4*, *LMOD1* and *ADAMTS19*, respectively (Fig. 2a–c).

### *Dtx4* and *Nav1* are dysregulated during complete UUO in vivo

**Fig. 3:**
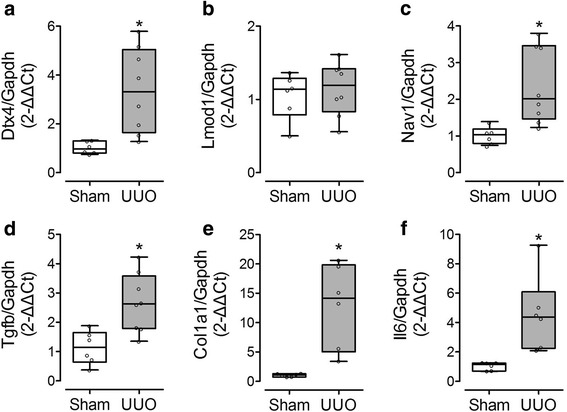
Selected mRNA predicted target expression in response to adult complete UUO in vivo. Renal expression of E3 ubiquitin-protein ligase DXT4 (Dtx4) (**a**), leiomodin-1 (Lmod1) (**b**), neuron navigator 1 (Nav1) (**c**), transforming growth factor beta (Tgfb) (**d**), collagen 1 (Col1a1) (**e**), and interleukin 6 (Il6) (**f**) was assessed by RT-PCR in adult mice after 7 days UUO.*p < 0.05 versus Sham

Since obstructive nephropathy potentially induces different pathways in the developing and the adult kidney we also verified the expression of the mRNA targets in the adult mouse complete UUO model, using eight male mice with complete UUO and six sham operated mice. We demonstrated that, similarly to what we observed in partial UUO in neonatal mice, *Dtx4* and *Nav1* expression was significantly increased in UUO mice compared to the sham (Fig. 3a and c), while *Lmod1* was not modified (Fig. 3b). The signal for *Adamts19* was too weak to provide any reliable data. In addition to the targets, we also showed that the renal expression of *Tgfβ* and collagen 1, two markers of fibrosis, and *IL-6*, a marker of inflammation was significantly increased in UUO mice compared to the sham (Fig. 3d–f), validating the pathological changes observed in this model.

## Discussion

Obstructive nephropathies, with UPJ obstruction as the prototypic obstructive nephropathy, are frequently encountered developmental anomalies in the pediatric population. Although renal fibrosis and inflammation are involved in the pathophysiology of severe UPJ obstruction, the molecular mechanisms underlying evolution of the lesions remain undetermined. In addition, most of this information has been obtained in animal models of disease. Recently discovered small non-coding RNAs called miRNAs are excreted and are stable in urine. Hence, urinary miRNAs could help to further decipher the pathophysiology of UPJ obstruction and generate the missing link between human and animal data. In this study we combined human urinary miRNA data and animal kidney tissue miRNA and mRNA data in a systems biology approach to obtain insight in the pathophysiology of the disease and improve upon the translatability of the results. The combined approach and independent validation pointed to the consistent dysregulation of specific miRNAs and to new potential targets in UPJ obstruction.

MiRNAs let-7a-5p, miR-125b-5p, miR-16-5p, miR-26a-5p and miR-29b-3p were consistently modified in mice and humans. Among these, miR-29b is a well-known player in renal pathologies and especially fibrosis. Indeed, miR-29b targets specific fibrotic molecules including collagens or α-smooth muscle actin, and its abundance is reduced in many fibrotic pathologies as its expression is inhibited by TGFβ [22, 27]. The protective role of miR-29b in fibrosis was further demonstrated in vivo since restoring miR-29b levels in a diabetic nephropathy animal model reversed accumulation of renal extracellular matrix [28]. On the other hand, to our knowledge, this is the first time that miR-16 and let-7a are associated to the development of kidney disease, as these miRNAs were mostly characterized to be involved in cancer [29–34]. Downregulation of let-7a has also been observed in scleroderma, contributing to the excessive deposition of collagen and tissue fibrosis in the skin [35]. Few data is available for miR-125b and kidney disease. Circulating miR-125b was found downregulated in chronic kidney disease (CKD) in hypertensive patients suffering from cardiovascular disease [36]. In another study upregulation of miR-125b protected against cisplatin-induced kidney injury via inhibition of Nuclear factor erythroid-2-related factor 2 [37]. MiR-26a has previously been reported to be over expressed in lung epithelia during development and to be involved in glomerular and tubular injury related to podocyte damage and maintenance of glomerular filtration rate [38]. A recent small-scale study (*n* = 4 UPJ obstruction patients vs *n* = 4 tumor-resection controls) identified in kidney tissue five miRNAs associated to UPJ obstruction. None of those overlapped with the human urinary miRNAs in our study and one, miR-342-5p, was also found in kidney tissue to be associated to obstruction in the partial neonatal mouse model, however with an opposite regulation [39].

A total of 35 putative mRNA targets for these differentially expressed miRNAs were predicted with the use of Ingenuity Pathway Analysis. In vitro validation of the predicted miRNA-mRNA pairs and in vivo assessment of the observed mRNA changes led to the sound confirmation of the regulation of E3 ubiquitin-protein ligase DTX4 by let-7a. Although E3 ubiquitin-protein ligase DTX4 has not yet been found associated to obstructive nephropathy, using the Kidney & Urinary Pathway Knowledge Base (KUPKB [40],) we observed that it has been found to be induced in models of polycystic kidney disease [41, 42] and in a lupus nephritis model [43]. Furthermore, DTX4 is a member of Notch Signaling non-canonical pathway [44]. It has been described that activation of Notch signaling can lead to fibrosis [45–49]. Hence one can hypothesize that downregulation of let-7a, activates DTX4 and the Notch signaling pathway, promoting the progression of fibrosis in UPJ obstruction.

NAV1 is a protein mostly found in neurons and is reported to play a critical role in microtubule development [50], but has not yet been implicated in UPJ obstruction. Coinciding with DTX4, NAV1 expression is also induced in a model of polycystic kidney disease [41] and is induced in vitro by the major pro-fibrotic cytokine TGFβ [51], potentially linking NAV1 induction to fibrosis. It is notable, though, that the regulation of NAV1 by miR-29b-3p did not follow the classical regulation pattern (up regulation of a miRNA causes down regulation of a target or *vice versa*) in the partial UUO model. In contrast, in the in vitro experiment NAV1 followed the predicted regulation and confirming that it may be a direct target of miR-29b. Previous reports have documented that some miRNAs may not target mRNAs directly, but only block the protein translation, leaving the mRNA intact [52–55]. Furthermore, other reports point to the fact that miRNAs may induce the same direction of regulation of their mRNA targets depending on the timeframe and conditions [56–60], providing an explanation of a possible indirect mechanism of miR-29b-NAV1 regulation. Nevertheless, further investigation is needed to determine if NAV1 is a direct target of miR-29b in vivo and if it is an interesting molecule in the context of UPJ obstruction. Combination of the human urine miRNA data with the mouse tissue data increased the confidence in the human data and allowed selecting the most promising miRNAs in human disease. A downside of our approach is that this type of prioritization will only focus on part of the molecular mechanisms of UPJ. If signals for specific mechanisms are absent in, for example, humans due to technical limitations or absence of shedding of miRNAs in urine but not in mouse tissue, this mechanism is not necessarily irrelevant in the pathophysiology of UPJ. Another shortcoming of our study is the fact that we used unadjusted *p*-values. Infants with UPJ obstruction included in the study displayed a wide range of hydronephrosis (grade 1–4, Table 1) and pelvic diameter (6–40 mm, Table 1) which generates considerable variability in the UPJ obstruction group. The mean fold change after comparison of healthy controls and UPJ obstruction patients was only 1.21 fold (±0.27) in this data. This is probably due to the fact that during excretion/shedding of miRNAs in urine the in-situ miRNA changes are flattened out. Moreover, correction for multiple testing resulted in no significant miRNAs in humans. We therefore continued the prioritization with unadjusted *p* values but we compensated for this shortcoming by the independent validation of the selected targets. Finally, the question remains whether observations in mouse tissue or human urine are faithfully reflecting the changes in the human kidney. In the past, studies have used a similar approach as ours, i.e. by combining urine and tissue analysis, and/or by combining human and animal data, to understand the role of miRNAs in the development of renal lesions. For example, miR-21 has been found to be consistently increased in animal and human samples of both urine and kidney tissue in the context of renal fibrosis [18, 61–63]. However, during acute kidney injury, miR-21 was increased in kidney tissue and decreased in the urine in a rat model, and increased in human urine [64]. In a study of Wang et al. again in a kidney injury model, miR-10a and miR-30d were found decreased in tissue and increased in urine after injury [65]. Finally, miR-192 was observed in different studies to be up-regulated in tissue in animal model of fibrosis, but down-regulated in human tissue samples and increased in human urine samples [66–69]. These examples show that there is no common regulation pattern for miRNAs in urine and kidney and in different species, even in the same pathological context and despite the fact that their role in renal lesions has been established with confidence. But by taking advantage of the conservation ability of miRNAs, combination of urine and tissue analysis, and/or human and animal data allows to identify features that are consistently and significantly modified as being associated with the disease with more confidence and can help transfer observations from animal models to human research.

In our study, we did not observe enriched canonical pathways when studying the miRNAs of human or mouse UPJ obstruction separately even if around hundreds of miRNAs were found significantly different. Hence we have used both statistical selection, combination of different omics data (miRNA and mRNA data) and pathway enrichment analysis to identify miRNAs and their targets most likely involved in the etiology of UPJ obstruction.

## Conclusion

Collectively this study is the first to correlate a mouse model of neonatal partial UUO with human UPJ obstruction in a comprehensive systems biology analysis. Our data revealed let-7a and miR-29b as molecules potentially involved in the development of fibrosis in UPJ obstruction via the control of DTX4 in both man and mice that would not be identified otherwise. We believe that our approach of combining omics data is generally applicable. Many omics studies generate long lists of differentially expressed molecules that are difficult to prioritize and does not necessarily inform on the actual impact of these changes in disease. To further improve on the validity and clinical translatability of the data, and because urine is a rich source of biomarkers that can be collected easily and non-invasively, combination of human urine and animal tissue data (e.g. miRNA, metabolites, proteins etc.) could be of great help to better understand the molecular mechanisms involved in the development of many complex diseases.

## Methods

### Human samples

#### Participants and urine collection

The studies were performed in accordance with the ethical principles in the Declaration of Helsinki and Good Clinical Practice and was approved by the CPP SOOM II (number DC-2008-452). Written informed consent was obtained from the parents of all child participants. The UPJO group was composed of patients with grade 1 to 4 hydronephrosis and a renal pelvic diameter between 6 and 40 mm (Table 1). Urine was collected from boys (<1 year). Urine from patients was collected in the morning during 30 min using a sterile pediatric urine collection pouch (B. Braun, Boulogne, France) during hospital consultation. Samples from healthy controls were both collected in a hospital setting (from newborns with heart murmur) and at home. Immediately after collection, all urines were frozen at −20 °C and stored at −80 °C.

### Mouse models

All mouse experiments were conducted in accordance with the NIH guide for the care and use of laboratory animals and were approved by the University of Virginia Animal Care and Use Committee (for the neonatal partial obstruction model) and the animal care and use committee UMS US006/INSERM, Toulouse, France (protocol number CEEA-122 2014-06/02605.01) for the complete adult obstruction model.

Both the partial neonatal and adult complete unilateral ureteral obstruction (UUO) mouse models have been previously described [70, 71]. Neonatal mice and adult mice were sacrificed after 5–7 and 7 days of obstruction, respectively.

### Microarray analysis

Microarray analysis was carried out as described previously [72, 73]. Briefly, total RNA was extracted from kidney tissues of nine neonatal mice with partial UUO (hydronephrosis grades 2 and 3) and nine control sham operated mice. Fluorescently labeled cRNA targets were generated using the Fluorescent Linear Amplification Kit (Agilent) and 10.0 mM Cyanine 3- or 5-labeled CTP (PerkinElmer, Boston, MA), and following user’s manual. Cyanine labeled cRNA from UUO kidneys was mixed with the same amount of reverse-color cyanine-labeled cRNA from the corresponding control kidney samples and hybridized on the Agilent 44 K Mouse Whole Genome Oligonucleotide Microarray. Array hybridization, washing and scanning were performed as previously described [72, 73].

MicroRNA expression profiling was performed using the Mouse miRNA Microarray Release 15.0 (8 × 15 K, G4471A-029152, Agilent Technologies), and the Human miRNA V3 Microarray Release 12.0 (8 × 15 K, G44710C-021827, Agilent Technologies). Briefly, 100 ng of total RNA was labeled and hybridized using the commercial miRNA Microarray System with miRNA Complete and Hybridization Kit (Agilent Technologies) following manufacturer’s instructions. Array hybridization, washing, scanning, data extraction, and analyses were performed as described previously.

### Cell models and antagomir treatment

Human HK-2 cells were grown in a DMEM/F-12 Nut Mix medium supplemented with 10% heat-inactivated fetal calf serum (FCS; GIBCO, Grand Island, NY, USA), 10 μg/mL of EGF, 5 μg/mL of insulin, 4 pg/mL Triiodothyronine (T3), 36 ng/mL of hydrocortisone. After 24 h of FCS starvation, HK-2 cells were transfected with 5 nmol IDT^®^ miRNA inhibitor targeting five miRNAs: hsa-let-7a-5p (ref. no 67488991), hsa-miR-125b-5p (ref. no 67488990) hsa-miR-16-5p (ref no. 67488992), hsa-miR-26a-5p (ref. no. 67488993) and hsa-miR-29b-3p (ref. no. 67488994), or with scrambled siRNA (Integrated DNA Technologies, Leuven, Belgium), using the DharmaFECT Duo transfection reagent (Dharmacon, Lafayette, CO, USA).

### Gene expression analysis

Total RNA was extracted from human kidney cells (HK2 cells) and complete UUO kidney samples using the Illumina’s Epicentre MasterPure Kit (Madison, WI, USA). Reverse transcription was performed for the miRNAs with MiRCURY LNA Universal RT Kit of Exiqon (Vedbaek, Denmark) and for the mRNA with High Capacity cDNA Reverse Transcription Kit of Thermo Scientific (Waltham, MA, USA) on a FlexCycler2 (Analytik Jena AG, Jena, Germany). Quantitative PCR amplification was performed on a StepOnePlus Real-Time PCR System (Thermo Scientific Waltham, MA, USA). Sybr Green technology was used for miRNAs according to Exiqon’s kit, while for the mRNA PCR the MESA BLUE qPCR MasterMix Plus kit from Eurogentec (Liege, Belgium). The primers used for the PCR are listed in Additional file 9.

### Bioinformatic analysis

Network and pathway analysis of the microarray data were performed using Ingenuity Pathway Analysis (IPA) software version 18488943 (IPA^®^, QIAGEN Redwood City, see http://www.Ingenuity.com).

### Statistics

For the microarrays statistical analysis the freely available software Gene ARMADA was used [74]. A background correction was made for all arrays by loess correction and normalization was made by linear lowess followed by quantile normalization. The statistically different genes were the result of a *t*-test analysis (*p*-value < 0.05). The results of the qPCR for the cell cultures and the partial and complete UUO experiments were expressed in fold change units based on 2^-ΔΔCt^ method. The graphs and the statistical analysis (Mann-Whitney test between the groups with *p*-value < 0.05 as significant) were performed with GraphPad Prism v5.0.

## Additional files

Additional file 1: Differentially expressed miRNAs in human UPJ. (XLSX 15 kb)
Additional file 2: Differentially expressed miRNAs in mouse partial UUO. (XLSX 11 kb)
Additional file 3: Differentially expressed mRNAs in mouse partial UUO. (XLSX 44 kb)
Additional file 4: Common dysregulated miRNAs between human UPJ (urine) and mouse partial UUO (kidney tissue). (XLSX 9 kb)
Additional file 5: miRNAs and their respective mRNA targets. (XLSX 12 kb)
Additional file 6: Molecular network predicted by IPA including let-7a-5p, miR-125b-5p and miR-16-5p and possibly related to hematological and immunological disease. Red = up regulated green = down regulated; all colored genes were identified in the partial UUO mouse mRNA dataset. (JPG 355 kb)
Additional file 7: Molecular network predicted by IPA including miR-26a-5p and miR-29b-3p and possibly related to organismal Injury and abnormalities. Red = up regulated green = down regulated; all colored genes were identified in the partial UUO mouse mRNA dataset. (JPG 318 kb)
Additional file 8: Molecular network predicted by IPA including miR-29b-3p and possibly related to cell death and survival and connective tissue development and function. Red = up regulated green = down regulated; all colored genes were identified in the partial UUO mouse mRNA dataset. (JPG 406 kb)
Additional file 9: Sequences of primers used. (DOCX 11 kb)

## Abbreviations

CAKUT: Congenital anomalies of the kidney and the urinary tract
CKD: Chronic kidney disease
DTX4: E3 ubiquitin-protein ligase
ESRD: End stage renal disease
HK2: Human kidney cells
IPA: Ingenuity Pathway Analysis
KUPKB: Kidney & Urinary Pathway Knowledge Base
NAV1: Neuron navigator 1
UPJ: Ureteropelvic junction
UUO: Unilateral ureteral obstruction
