# Supplementary figures and images for: Systems biology combining human- and animal-data miRNA and mRNA data identifies new targets in ureteropelvic junction obstruction
Source: BMC Syst Biol. 2017 Mar 1;11:31. doi: 10.1186/s12918-017-0411-7 (PMC5333413; doi:10.1186/s12918-017-0411-7)

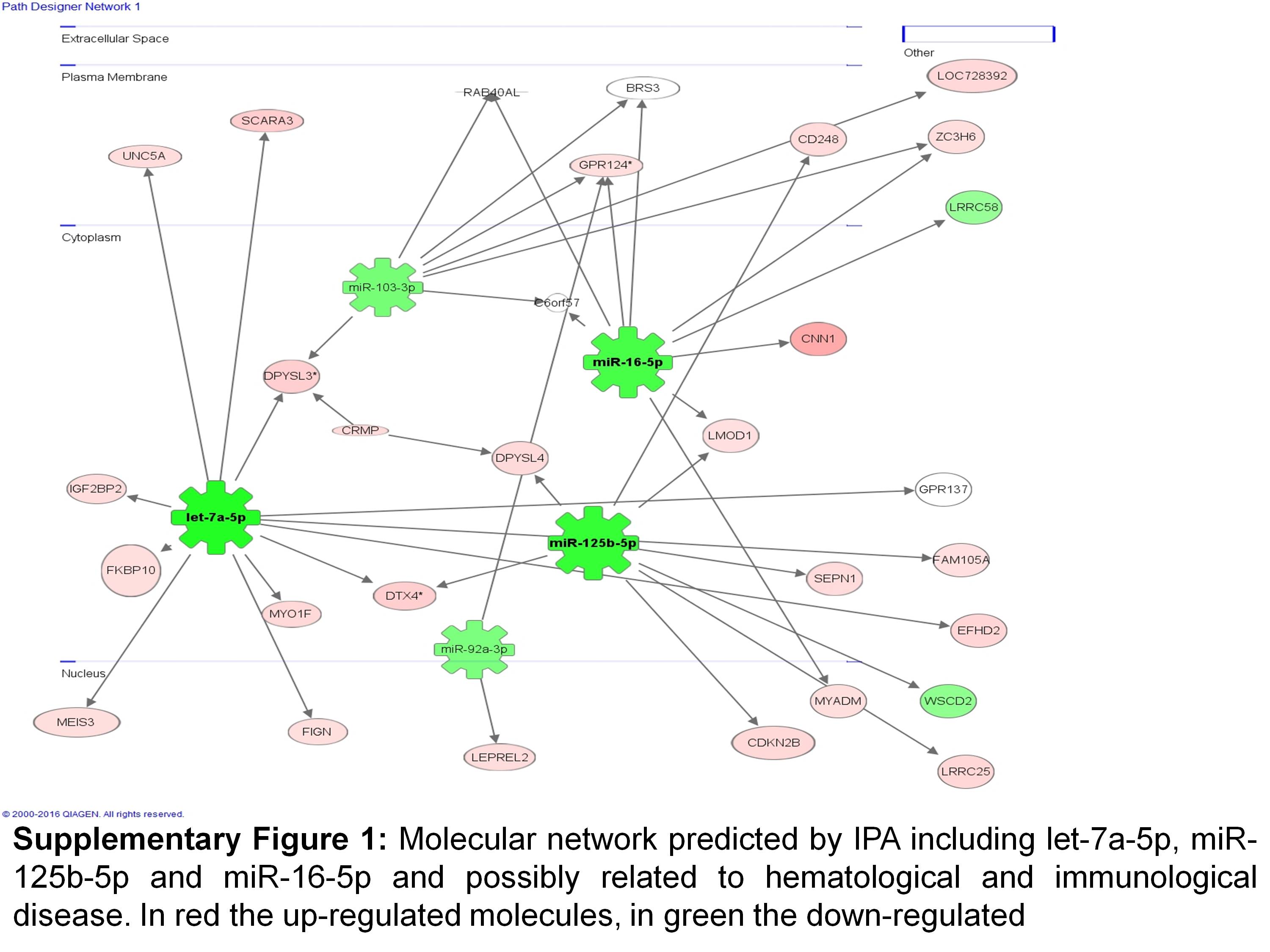

Supplement: Additional file 6: — Molecular network predicted by IPA including let-7a-5p, miR-125b-5p and miR-16-5p and possibly related to hematological and immunological disease. Red = up regulated green = down regulated; all colored genes were identified in the partial UUO mouse mRNA dataset. (JPG 355 kb) [file 12918_2017_411_MOESM6_ESM.jpg]

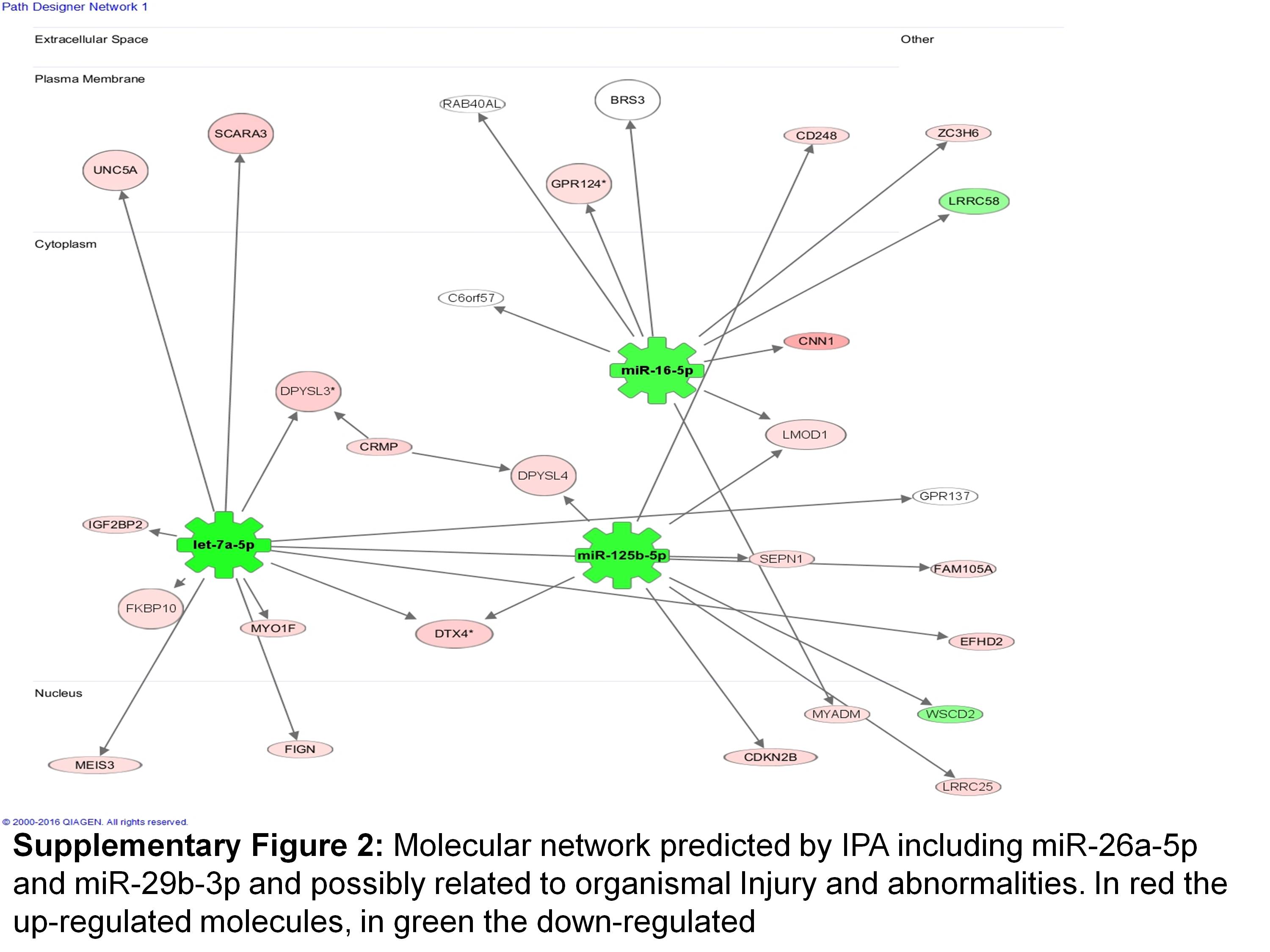

Supplement: Additional file 7: — Molecular network predicted by IPA including miR-26a-5p and miR-29b-3p and possibly related to organismal Injury and abnormalities. Red = up regulated green = down regulated; all colored genes were identified in the partial UUO mouse mRNA dataset. (JPG 318 kb) [file 12918_2017_411_MOESM7_ESM.jpg]

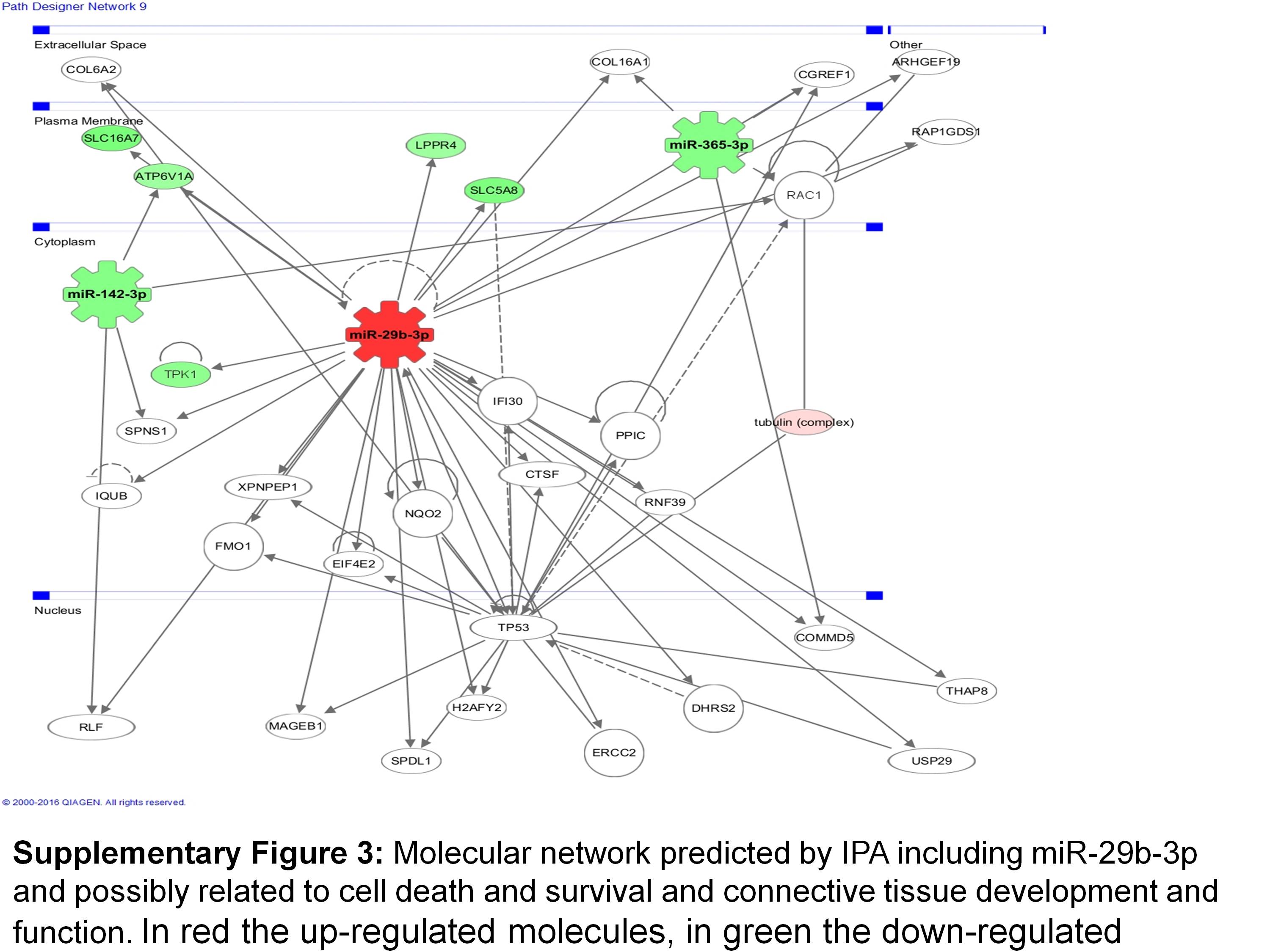

Supplement: Additional file 8: — Molecular network predicted by IPA including miR-29b-3p and possibly related to cell death and survival and connective tissue development and function. Red = up regulated green = down regulated; all colored genes were identified in the partial UUO mouse mRNA dataset. (JPG 406 kb) [file 12918_2017_411_MOESM8_ESM.jpg]
