# Supplementary material for: Systems biology combining human- and animal-data miRNA and mRNA data identifies new targets in ureteropelvic junction obstruction
Source: BMC Syst Biol. 2017 Mar 1;11:31. doi: 10.1186/s12918-017-0411-7 (PMC5333413; doi:10.1186/s12918-017-0411-7)
Supplement: Additional file 9: — Sequences of primers used. (DOCX 11 kb) [file 12918_2017_411_MOESM9_ESM.docx]

Supplementary Material

Primers used for RT-qPCR

Primer sequences for miRNAs were obtained for hsa-let-7a-5p (ref.no. 205727), hsa-miR-125b-5p (ref. no. 205713), hsa-miR-16-5p (ref. no. 205702), hsa-miR-26a-5p (ref.no. 206023) and hsa-miR-29b-3p (ref. no. 204679) from Exiqon (Vedbaek, Denmark).

Primers for mRNAs quantification were obtained from Integrated DNA Technologies (Leuven, Belgium):

mouse DTX4:

m-DTX4-F: ACATCTACTGCCTGGTGGCTATG

m-DTX4-R: GGGCACTGTAAACTCCCATCCTTG

mouse Nav1:

m-Nav1-F: ACCCAAGGGAATGATTCGGTCAG

m-NAV1-R: ACAGCACCGAGCCATGAACATC

mouse LMOD1:

m-LMOD-F: AGATGTCTGTGGATGAAAGCAAGC

m-LMOD1-R: TCCTCTCCATTCTTGGCATCTGTC

mouseADAMTS19:

m-ADAMTS19-F: GCGAGTAGGTGACTGGTCTAAGTG

m-ADAMTS19-R: ACACGAGACTGCATGCCTTTGC

mouse LRRC58:

m-LRRC58-F: TCCAAACCCAAAGTGTGGTGGA

m-LRRC58-R: GGAGAGCACAGATAGTGCATGAGG

mouse-GAPDH:

m-GAPDH-F: CTTTGTCAAGCTCATTTCCTGG

m-GAPDH-R: TCTTGCTCAGTGTCCTTGC

mouse TGFb1:

m-TGFb1-F: CCTGAGTGGCTGTCTTTTGA

m-TGFb1-R: CGTGGAGTTTGTTATCTTTGCTG
